# Chromosomal instability as a prognostic marker in cervical cancer

**DOI:** 10.1186/s12885-015-1372-0

**Published:** 2015-05-06

**Authors:** Christine How, Jeff Bruce, Jonathan So, Melania Pintilie, Benjamin Haibe-Kains, Angela Hui, Blaise A Clarke, David W Hedley, Richard P Hill, Michael Milosevic, Anthony Fyles, Fei-Fei Liu

**Affiliations:** 1 Princess Margaret Cancer Centre, University Health Network, Toronto, ON Canada; 2 Department of Radiation Oncology, Princess Margaret Cancer Centre, University Health Network, Toronto, ON Canada; 3 Department of Radiation Oncology, University of Toronto, Toronto, ON Canada; 4 Division of Biostatistics, Princess Margaret Cancer Centre, University Health Network, Toronto, ON Canada; 5 Department of Pathology, University Health Network, Toronto, ON Canada; 6 Division of Medical Oncology, Princess Margaret Cancer Centre, Toronto, ON Canada; 7 Medical Biophysics Department, University of Toronto, Toronto, ON Canada

**Keywords:** Cervical cancer, Chromosomal instability, CIN, mRNA, TCGA

## Abstract

**Background:**

Cervical cancer is the third most common cancer in women globally, and despite treatment, distant metastasis and nodal recurrence will still develop in approximately 30% of patients. The ability to predict which patients are likely to experience distant relapse would allow clinicians to better tailor treatment. Previous studies have investigated the role of chromosomal instability (CIN) in cancer, which can promote tumour initiation and growth; a hallmark of human malignancies. In this study, we sought to examine the published CIN70 gene signature in a cohort of cervical cancer patients treated at the Princess Margaret (PM) Cancer Centre and an independent cohort of The Cancer Genome Atlas (TCGA) cervical cancer patients, to determine if this CIN signature associated with patient outcome.

**Methods:**

Cervical cancer samples were collected from 79 patients, treated between 2000–2007 at the PM, prior to undergoing curative chemo-radiation. Total RNA was extracted from each patient sample and analyzed using the GeneChip Human Genome U133 Plus 2.0 array (Affymetrix).

**Results:**

High CIN70 scores were significantly related to increased chromosomal alterations in TCGA cervical cancer patients, including a higher percentage of genome altered and a higher number of copy number alterations. In addition, this same CIN70 signature was shown to be predictive of para-aortic nodal relapse in the PM Cancer Centre cohort.

**Conclusions:**

These findings demonstrate that chromosomal instability plays an important role in cervical cancer, and is significantly associated with patient outcome. For the first time, this CIN70 gene signature provided prognostic value for patients with cervical cancer.

## Background

Chromosomal instability (CIN), a condition in which cells change their chromosomal content at a high rate, is a consistent feature of the majority of solid tumours [1,2]. It has long been postulated that chromosomal imbalance plays a role in tumourigenesis, since aneuploid karyotypes were first observed in cancer cells over a century ago [3]. Since then, evidence has shown that CIN promotes tumour initiation and growth [4-7]. In patient tumours, it has been demonstrated that CIN increases with increasing tumour grade as well as invasiveness [8-11]. Some studies have alluded to the clinical value of CIN in human cancers [8,12], although therapeutic targeting of CIN remains in its infancy [13].

Using a computational approach to identify specific genes whose expression was consistently correlated with total functional aneuploidy across multiple cancer types, Carter *et al.* developed a gene expression signature of CIN, the CIN70, which could predict patient survival and prognosis [14]. Over-expression of this CIN70 signature was predictive of poor clinical outcome in 12 datasets representing six types of tumour: lymphoma, lung adenocarcinoma, glioma, medulloblastoma, mesothelioma, and breast cancer [15-26]. In this study, we sought to examine CIN in cervical cancer and determine if the CIN70 signature could also be used to predict clinical outcome in patients with cervical cancer.

Globally, cervical cancer is the third most common cancer in women [27]. Although there has been a decrease in cervical cancer incidence and mortality over the past thirty years in the United States, the five-year survival rate remains below 40% for stage III and stage IV patients [28]. Furthermore, distant metastasis and lymph node recurrence occurs in approximately 30% of patients following primary treatment [29]. The ability to predict which patients are likely to experience distant relapse would allow clinicians to better tailor patient therapy.

In this current study, the CIN70 signature was investigated in a cohort of cervical cancer patients treated at the Princess Margaret (PM) Cancer Centre (n = 79), and an independent cohort of TCGA cervical cancer patients (n = 130). CIN70 score was found to be significantly associated with chromosomal alterations and para-aortic distant relapse in patients. Altogether, these findings provide insight into the role of CIN in cervical cancer and show that CIN can harbour clinical value for patients.

## Methods

### Ethics statement

Written informed consent was obtained from all human subjects, according to a protocol (09-0245-T) approved for this study by the University Health Network Research Ethics Board.

### Clinical specimens

Frozen pre-treatment cancer samples were collected from 79 patients with cervical cancer, prior to undergoing curative chemo-radiation, consisting of external-beam radiotherapy to the primary cervical tumour and pelvic lymph nodes (45 to 50 Gy total, in 1.8 to 2 Gy daily fractions using 18 or 25 Megavolt photons), combined with weekly cisplatin (40 mg/m^2^ total, 5 doses). These patients were treated at the PM Cancer Centre between 2000 and 2007. Patients were staged using the FIGO (International Federation of Gynecologists and Obstetricians) system, with additional clinical information gathered using computed tomography (CT) scans of the abdomen and pelvis, as well as magnetic resonance imaging (MRI) of the pelvis to assess local and lymphatic disease. Pelvic and para-aortic lymph nodes were classified as positive for metastatic disease if the MRI short-axis dimension was >1 cm, and equivocal if it was between 8 to 10 mm.

The frozen biopsy specimens were placed in a storage medium (optimal cutting temperature (OCT) compound) for histopathologic examination, then flash-frozen in liquid nitrogen. H&E-stained tissue sections were cut from the OCT-embedded material, and evaluated by a gynecology oncology pathologist (B. Clarke). The total cell content (stroma and tumour cells) was estimated for all tissue samples using a light microscope, and only samples containing at least 70% tumour cells were considered for further analysis. Flash-frozen normal cervix tissues obtained from 11 patients who underwent total hysterectomy for benign causes served as the normal comparators.

### Sample processing

Two sections of 50-μm thickness were cut from the OCT-embedded flash-frozen tissues and placed in a nuclease-free microtube. Total RNA was isolated using the Norgen Total RNA Purification Kit (Norgen Biotek), according to the manufacturer’s instructions. Gene expression was measured with the GeneChip Human Genome U133 Plus 2.0 array (Affymetrix).

### Data analysis

Affymetrix array data were pre-processed using the Robust Multi-array Average robust-multi array algorithm [30] in the R statistical environment with the affy package [31]. CIN70 score was calculated by summing the normalized expression of each gene in the CIN70 signature. For genes with more than one mapped probe set on the array, the probe set with the highest variance across samples was selected.

Level 3 copy-number (SNP 6.0 arrays; Affymetrix), gene-expression (RNA-Seq; Illumina) and somatic mutation (Exome-Seq; Illumina) data were downloaded from the Broad GDAC Firehose (http://gdac.broadinstitute.org/), and analyzed in the R statistical environment. CIN70 score was calculated again by summing the normalized expression of each gene in the CIN70 signature. The number of copy-number alterations was calculated using segmented copy-number data, whereby segments with a mean log2 copy-number ratio value >0.2 or < −0.2 were deemed altered [32,33]. Relatedly, percent genome altered was calculated by adding the length of each “altered” segment, divided by the total length of the genome analyzed. The number of mutations corresponded to somatic coding mutations were called using TCGA’s Exome-Seq analysis pipeline.

### Survival analysis

For each patient in the PM Cancer Centre cohort (n = 79), a risk score was calculated using the published CIN70 signature [14] and Affymetrix gene expression data. Risk scores were dichotomized at the median (CIN70 = 7.57) and the cohort was divided into low and high-risk groups. Curves for overall survival (OS), disease-free survival (DFS), probability of local relapse, and probability of distant relapse were plotted according to the Kaplan-Meier method, with p-values determined using the Wald test.

## Results

### PM and TCGA cohorts showed a distinct expression pattern of CIN70 genes according to CIN70 score

**Figure 1:**
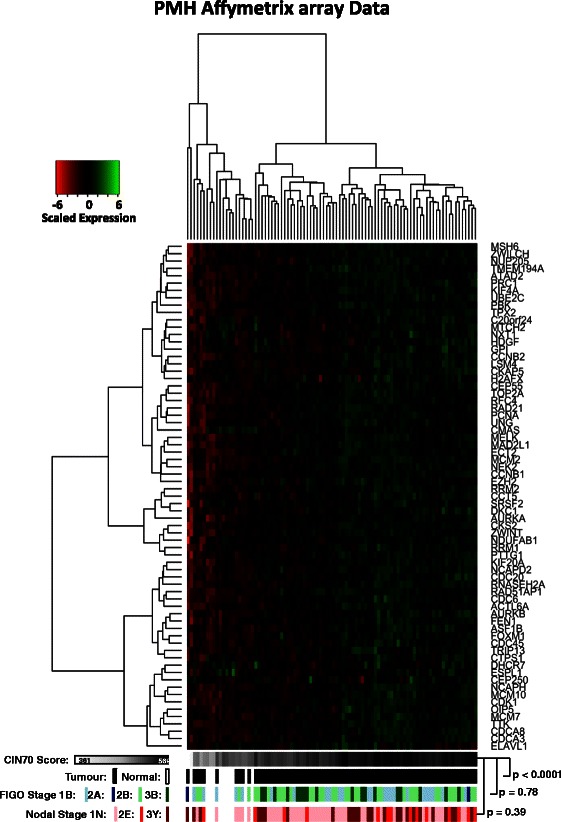
PM Cancer Centre Affymetrix heat map. Hierarchically clustered heat map showing scaled expression of CIN70 genes in cervix tumour (n = 79) and normal (n = 11) tissues, compared to CIN70 score (white to black scale). Comparisons are also made with FIGO stage (1B, 2A, 2B, and 3B), and nodal stage (1 N = negative, 2E = equivocal, 3Y = positive). P-values refer to relationship between CIN70 scores with tumour:normal, FIGO stage, and Nodal stage.

**Figure 2:**
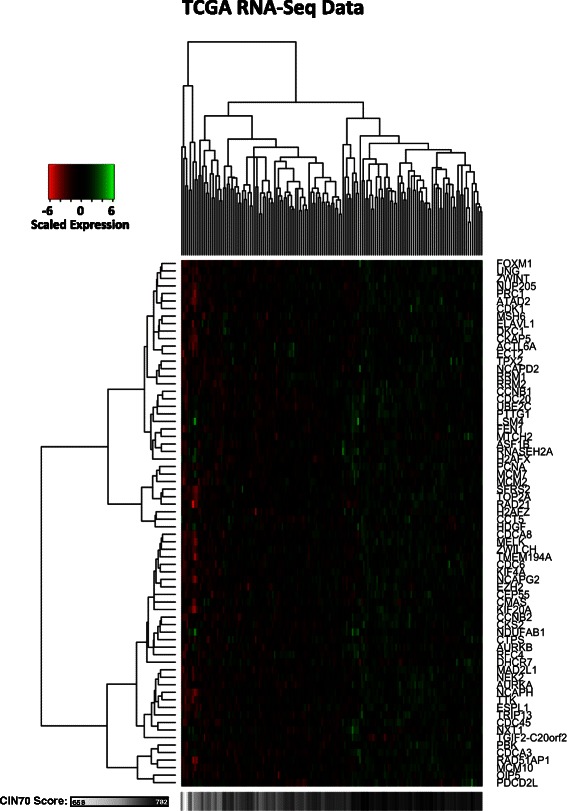
TCGA RNA-Seq heat map. Hierarchically clustered heat map showing scaled expression of CIN70 genes in TCGA cervix tumour tissues (n = 130), compared to CIN70 score.

The clinical characteristics of the 79 PM Cancer Centre and 130 TCGA patients are shown in Tables 1 and 2, respectively. A heat map of scaled expression of the CIN70 genes showed a distinct expression pattern in patients according to CIN70 score (Figure 1). As expected, normal and tumour cervix samples had significantly different CIN70 scores (p < 0.0001). Interestingly, CIN70 score was not significantly associated with FIGO stage (p = 0.78) or nodal stage (p = 0.39). TCGA cervical cancer patients showed a similar expression pattern of the CIN70 genes according to CIN70 score (Figure 2).

**Table 1:** **Clinical parameters of the Princess Margaret Cancer Centre cohort**

	*n = 79*
**Age (years)**	
Median	48
Range	26-84
**Tumour size**	
≤ 5 cm	48 (61%)
> 5 cm	31 (39%)
**FIGO stage**	
IA	0
IB	24 (30%)
IIA	2 (3%)
IIB	35 (44%)
IIIA	0
IIIB	18 (23%)
**Pelvic or para-aortic node involvement**	
Positive	25 (32%)
Equivocal	15 (19%)
Negative	39 (49%)
**Overall survival**	
Deaths	24 (31%)
**Disease-free survival**	
Relapses or deaths	28 (35%)
**Follow-up (years)**	
Median	6.0
Range	0.7-10.6

**Table 2:** **Clinical parameters of TCGA cohort**

	*n = 135*
**Age (years)**	
Median	46
Range	21-88
**FIGO stage**	
IA	2 (1.5%)
IB	82 (60.7%)
IIA	11 (8.2%)
IIB	11 (8.2%)
IIIA	0
IIIB	19 (14.1%)
IVA	1 (0.7%)
IVB	3 (2.2%)
N/A	6 (4.4%)
**Overall survival**	
Deaths	19 (14%)
**Follow-up (years)**	
Median	0.36
Range	0-14.7

### High CIN70 score was related to increased chromosomal alterations

**Figure 3:**
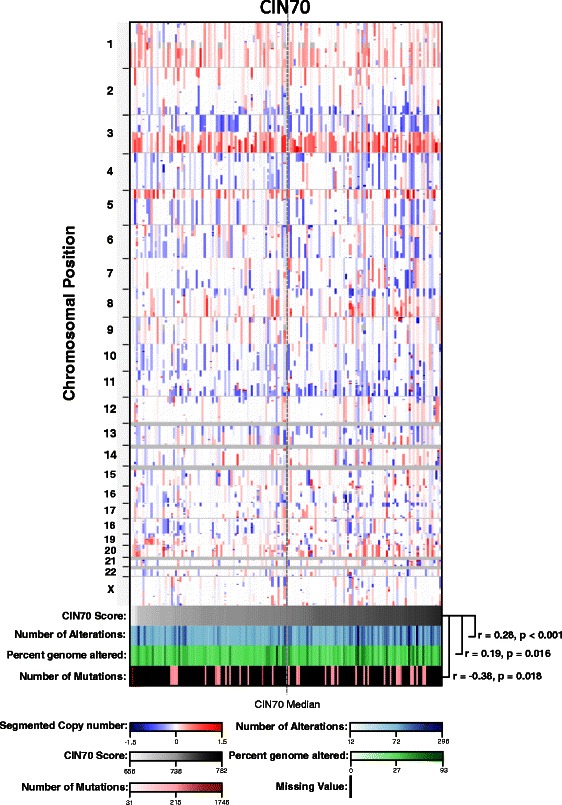
Chromosomal alterations in TCGA cervix cancer tissues. Copy number alterations (top) in TCGA cervical cancer patients, compared to CIN70 score (white to black scale), number of alterations (white to blue scale), percent genome altered (white to green scale), and number of mutations (white to red scale). Spearman’s correlation coefficient (r), and P-values are shown for each of the respective comparisons.

A heat map of TCGA patients (Figure 3) demonstrated the patterns of chromosomal alterations. Patients with a high CIN70 score (white to black scale) had a higher number of copy number alterations (white to blue scale; Spearman’s correlation coefficient (r) = 0.28, p < 0.001) and a higher percentage of genome altered (white to green scale; r = 0.19, p = 0.016). Interestingly, the number of mutations (white to red scale) was negatively correlated with the CIN70 score, whereby patients with higher CIN70 scores had fewer mutations (r = −0.38, p = 0.018); however, there were a significant number of patients with missing values for this parameter.

### CIN70 score was related to distant relapse in cervical cancer patients

**Figure 4:**
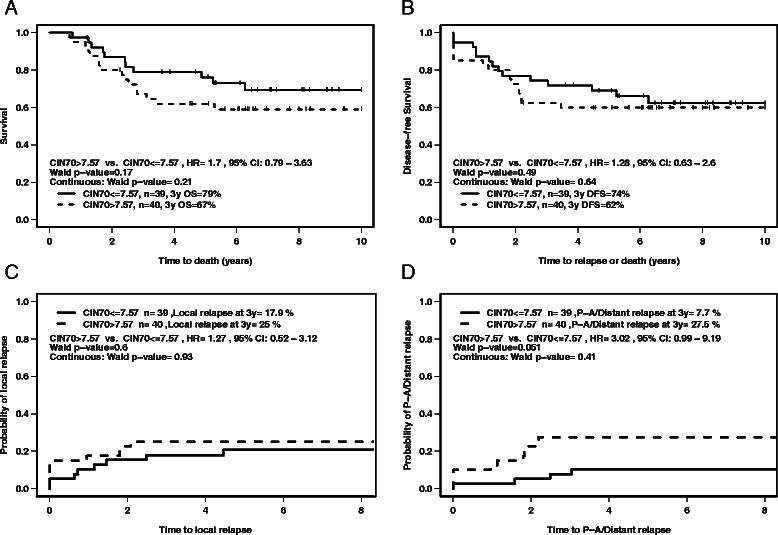
Kaplan-Meier plot of the 79 PM Cancer Centre cervical cancer patients according to CIN70 score. A risk score was calculated for each patient using the CIN70 signature. The median risk score was used to divide patients into high *vs.* low risk groups. Kaplan-Meier curves are shown for: **A)** overall survival; **B)** disease-free survival; **C)** local relapse; **D)** para-aortic or distant relapse. HR; hazard ratio, CI; 95% confidence interval, P-A; para-aortic.

The previously published CIN70 signature [14] was used to calculate the risk score for each patient (n = 79) and the median risk score was used to dichotomize a low *vs.* high-risk group. According to the Kaplan-Meier analysis, the CIN70 signature achieved borderline significance for para-aortic nodal or distant relapse (Figure 4D), with a hazard ratio of 3.02 and Wald p-value of 0.05, but not significant for OS, DFS, or local relapse (Figure 4A-C).

## Discussion

Previous studies have shown that CIN is a hallmark of most solid and many hematopoietic human cancers [2,8,10,11,34-37]. Various mechanisms of CIN have been described, including centrosome duplication [38-40], spindle assembly checkpoint defects [41,42], telomere dysfunction [43], faulty cell-cycle regulation [44-46], sister chromatid cohesion defects [47,48], and the regulation of microtubule attachments to chromosomes at kinetochores [49-51]. To date, CIN is rarely measured in cancer patients, due to technical complexity and lack of a clear understanding of the clinical value of CIN [12].

The CIN70 signature was developed by Carter *et al.* using a computational method to identify specific genes that were expressed in correlation with total functional aneuploidy, across multiple types of cancer [14]. Over-expression of this signature was shown to predict clinical outcome in six types of cancer, and was also able to stratify grade 1 and 2 breast tumours according to clinical outcome. Birkbak *et al.* reported that extremes of CIN score were associated with poor prognosis in four types of cancer (breast, ovarian, gastric, and non-small cell lung) [52]. Our study further extended the application of this CIN70 signature to cervical cancer. However, we failed to recapitulate the non-monotonic relationship of CIN with survival as reported in the Birbak *et al.* study. This could be due to the fact that our study was underpowered to detect a difference in survival, and we examined distant relapse instead of survival. Interestingly, we did observe an inverse relationship between CIN score/copy-number variation and the number of mutations in our cervical cancer samples. This confirms the reported data from Ciriello *et al.*, who analyzed global copy number variation and number of mutations across twelve different cancer types [53]. Our study, along with the others discussed above, are a step towards establishing CIN-associated molecular markers that can be measured in the clinic, and help expand the prognostic utility of CIN in a broad range of human malignancies. Further work should be conducted to determine if the CIN70 signature holds clinical value for other types of cancers, in addition to cervical cancer and the six cancers validated in the Carter *et al.* study.

## Conclusions

In summary, this study was the first to evaluate the previously published CIN70 signature in cervical cancer patients. CIN70 score was shown to be significantly associated with chromosomal alterations and para-aortic distant relapse. The Carter *et al*. study was the first step towards establishing CIN-associated molecular markers that could be measured in clinical specimens. Our study further extended the application of this CIN70 signature, and demonstrated that it was associated with para-aortic nodal, as well as distant relapse in patients with cervical cancer. Once longer follow-up is available for the TCGA cohort, it would be important to corroborate the prognostic value of this CIN70 signature for this independent group of patients.

## Abbreviations

CIN: Chromosomal instability
TCGA: The Cancer Genome Atlas
Gy: Gray
FIGO: International Federation of Gynecologists and Obstetricians
CT: Computed tomography
MRI: Magnetic resonance imaging
OCT: Optimal cutting temperature
H&E: Hematoxylin and eosin
OS: Overall survival
DFS: Disease-free survival
